# Genetic Evidence for Single-Strand Lesions Initiating Nbs1-Dependent Homologous Recombination in Diversification of Ig V in Chicken B Lymphocytes

**DOI:** 10.1371/journal.pgen.1000356

**Published:** 2009-01-30

**Authors:** Makoto Nakahara, Eiichiro Sonoda, Kuniharu Nojima, Julian E. Sale, Katsuya Takenaka, Koji Kikuchi, Yoshihito Taniguchi, Kyoko Nakamura, Yoshiki Sumitomo, Ronan T. Bree, Noel F. Lowndes, Shunichi Takeda

**Affiliations:** 1 CREST Research Project, Japan Science and Technology Agency, Saitama, Japan; 2 Department of Radiation Genetics, Graduate School of Medicine, Kyoto University, Kyoto, Japan; 3 Medical Research Council Laboratory of Molecular Biology, Division of Protein and Nucleic Acid Chemistry, Cambridge, United Kingdom; 4 Genome Stability Laboratory, Department of Biochemistry, National University of Ireland-Galway, Galway, Ireland; 5 National Centre for Biomedical Engineering Science, National University of Ireland-Galway, Galway, Ireland; Brandeis University, United States of America

## Abstract

Homologous recombination (HR) is initiated by DNA double-strand breaks (DSB). However, it remains unclear whether single-strand lesions also initiate HR in genomic DNA. Chicken B lymphocytes diversify their Immunoglobulin (Ig) V genes through HR (Ig gene conversion) and non-templated hypermutation. Both types of Ig V diversification are initiated by AID-dependent abasic-site formation. Abasic sites stall replication, resulting in the formation of single-stranded gaps. These gaps can be filled by error-prone DNA polymerases, resulting in hypermutation. However, it is unclear whether these single-strand gaps can also initiate Ig gene conversion without being first converted to DSBs. The Mre11-Rad50-Nbs1 (MRN) complex, which produces 3′ single-strand overhangs, promotes the initiation of DSB-induced HR in yeast. We show that a DT40 line expressing only a truncated form of Nbs1 (Nbs1^p70^) exhibits defective HR-dependent DSB repair, and a significant reduction in the rate—though not the fidelity—of Ig gene conversion. Interestingly, this defective gene conversion was restored to *wild type* levels by overproduction of *Escherichia coli* SbcB, a 3′ to 5′ single-strand–specific exonuclease, without affecting DSB repair. Conversely, overexpression of chicken Exo1 increased the efficiency of DSB-induced gene-targeting more than 10-fold, with no effect on Ig gene conversion. These results suggest that Ig gene conversion may be initiated by single-strand gaps rather than by DSBs, and, like SbcB, the MRN complex in DT40 may convert AID-induced lesions into single-strand gaps suitable for triggering HR. In summary, Ig gene conversion and hypermutation may share a common substrate—single-stranded gaps. Genetic analysis of the two types of Ig V diversification in DT40 provides a unique opportunity to gain insight into the molecular mechanisms underlying the filling of gaps that arise as a consequence of replication blocks at abasic sites, by HR and error-prone polymerases.

## Author Summary

An important class of chemotherapeutic drugs used in the treatment of cancer induces DNA damage that interferes with DNA replication. The resulting block to replication results in the formation of single-strand gaps in DNA. These gaps can be filled by specialized DNA polymerases, a process associated with the introduction of mutations or by recombination with an undamaged segment of DNA with an identical or similar sequence. Our work shows that diversification of the antibody genes in the chicken B cell line DT40, which is initiated by localized replication-stalling DNA damage, proceeds by formation of a single-strand intermediate. These gaps are generated by the action of a specific nuclease complex, comprising the Mre11, Rad50, and Nbs1 proteins, which have previously been implicated in the initiation of homologous recombination from double-strand breaks. However, in this context, their dysfunction can be reversed by the expression of a bacterial single-strand–specific nuclease, SbcB. Antibody diversification in DT40 thus provides an excellent model for studying the process of replication-stalling DNA damage and will allow a more detailed understanding of the mechanisms underlying gap repair and cellular tolerance of chemotherapeutic agents.

## Introduction

Homologous recombination (HR) contributes to genome maintenance by repairing double-strand breaks (DSBs) and single-strand lesions. It accomplishes this by associating the damaged DNA with intact homologous sequences (reviewed in [1]). Genetic studies of *Escherichia coli* indicate that DSBs are recognized by the RecBCD enzyme at the initial step of HR, while single-strand gaps are loaded with RecA with the help of the RecF, RecO and RecR (RecFOR) proteins [2] (reviewed in [3]). In yeast and vertebrate cells, however, it remains unclear whether single-strand lesions can also directly stimulate HR, or if their replication leads to DSBs, which then stimulate HR.

The process of DSB-induced HR is well characterized in the budding yeast [4]. First, DSBs are resected by a nuclease to generate a 3′ overhang. A major nuclease in this process is thought to be a complex containing three proteins: Mre11, Rad50 and Nbs1 (called the MRN complex) (reviewed in [5]). The role of the 3′–5′ exonuclease activity of purified Mre11 in DSB repair remains enigmatic, as DSB resection is of opposite polarity *in vivo* [6]. Recent studies indicate that the MRN complex requires another factor to function: CtIP, the ortholog of Sae2 and Ctp1 in *S. cerevisiae* and *S. pombe*, respectively [7]–[9]. Biochemical study demonstrated that Sae2, a cofactor of the MRN complex, can process a single strand nick, and expand it [10]. The single-strand DNA generated adjacent to the DSB is coated with polymerized Rad51, resulting in the formation of nucleoprotein filaments. The assembly of RAD51 at DNA damage sites is regulated by a number of RAD51 cofactors, including the tumor-suppressor gene BRCA1 (Breast Cancer Susceptibility Gene 1), BRCA2, and five RAD51 paralogs (RAD51B/C/D and XRCC2/3) (reviewed in [11],[12]). The Rad51-containing single-strand DNA filaments play a role in the search for homologous DNA sequences and subsequent strand invasion into homologous duplex DNA. The importance of the role of the MRN complex in genome maintenance is indicated by a marked increase in the number of spontaneously arising chromosomal breaks followed by cell death after depletion of Mre11 in DT40 cells [13], and is also indicated by the high incidence of tumorigenesis in certain hereditary diseases: ataxia-telangiectasia-like diseases (ATLD) and Nijmegen breakage syndrome (NBS), which result from hypomorphic mutations in the *MRE11* and *NBS1* genes, respectively [14]–[17].

A combination of HR and non-templated single-base changes contributes to Ig V sequence variation in chickens and in some mammalian species such as rabbits and cattle [18]. Similarly, the chicken DT40 B lymphocyte line undergoes templated HR-dependent diversification (hereafter called Ig gene conversion) as well as non-templated single-base substitutions (hereafter called Ig hypermutation) during *in vitro* passage [19]–[21]. HR introduces tracts of templated mutations to rearranged variable (V) regions [22]–[24]. An array of “pseudo-V_λ_” regions, located upstream from the functional rearranged VJ_λ_, provides donors for this non-reciprocal sequence transfer. Since donor and recipient segments have a ∼10% sequence divergence, sequential Ig gene conversion events are able to substantially diversify Ig V [24].

Both types of Ig V diversification are initiated by activation-induced deaminase (AID), which forms uracil from deoxycytidine (dC) [25]–[27]. Uracil is subsequently removed by uracil-DNA-glycosylase- (UNG) mediated hydrolysis, which generates abasic sites [28]–[30]. In *UNG^−/−^* DT40 cells, the rate of C to T transitions is more than ten times greater than in *UNG^+/+^* cells, indicating that more than 90% of the AID-induced uracil is accurately eliminated, presumably by base excision repair [28]. Non-templated hypermutation is generated as a consequence of translesion DNA synthesis (TLS) past abasic sites [31]. It is currently unclear how Ig gene conversion is induced by abasic sites, although it is likely that the abasic sites are converted to either single-strand gaps or DSBs, which in turn stimulate HR with upstream pseudo-V_λ_ segments. Current evidence points towards single-strand gaps, rather than DSBs, as the main downstream intermediate of abasic sites in the induction of Ig gene conversion for the following reason. In cells deficient in BRCA1, BRCA2 or Rad51 paralogs, where Rad51 is not accumulated efficiently at DNA lesions, the impaired HR causes a shift of Ig V diversification from HR- to TLS-dependent hypermutation [20],[32],[33]. Cleavage of template strands containing abasic sites cannot occur prior to TLS past the abasic sites. Thus, a common substrate for both Ig gene conversion and TLS is likely to be a single-strand gap and/or a stalled replication fork [34].

We hypothesized that if Ig gene conversion was triggered by single-strand lesions but not by DSBs, it would not involve the MRN complex (which is currently proposed as being involved in double-strand-break resection to generate recombinogenic 3′ ends). To test this hypothesis, we generated *nbs1* hypomorphic mutant DT40 cells, where Nbs1 null mutant cells were rescued by an *NBS1^p70^* transgene. The resulting *ΔNBS1/NBS1^p70^* cells shared a phenotype very similar to cell lines established from patients with Nigmegen-breakage syndrome [35], including significant reduction in the frequency of HR-dependent DSB repair. Unexpectedly, the defect of Nbs1 also suppressed Ig gene conversion by two orders of magnitude.

To further define the role of the MRN complex in Ig gene conversion, we next attempted to reverse the defective Ig gene conversion by ectopically overexpressing chicken Exo1 [36]–[40] or *E.coli* Exo1 (SbcB) [41]–[43]. Exo1 is an evolutionarily conserved double strand-specific 5′ to 3′ exonuclease, and involved in mismatch repair in the eukaryotic cells. Additionally, the eukaryotic Exo1 can promote HR by facilitating 3′ tail formation at DSBs [38],[39]. Although both eukaryotic Exo1 and SbcB expand single-strand gaps from single-strand breaks in mismatch repair, SbcB can digest single-strand DNA at an opposite direction, 3′ to 5′, and thereby suppress DSB induced HR by removing 3′ overhang at DSBs (reviewed in [3]). Remarkably, the ectopic expression of SbcB normalized Ig gene conversion, but overexpression of chicken Exo1 did not. Conversely, the ectopic expression of chicken Exo1, but not SbcB, increased the frequency of DSB-dependent gene-targeting [44],[45], presumably by promoting the resection of DSBs. These data argue against the possibility that SbcB promotes Ig gene conversion by processing DSBs. Hence, these data support the notion that single-strand gaps may be supported a common direct precursor of both Ig gene conversion and error-prone gap-filling. In addition, our study thus suggests that the MRN complex is involved in HR, probably in two different ways: by processing DSBs and by generating recombinogenic single-strand lesions.

## Results

### Generation of Nbs1^p70^-Expressing Clones and Nbs1-Null–Deficient Cells

**Figure 1:**
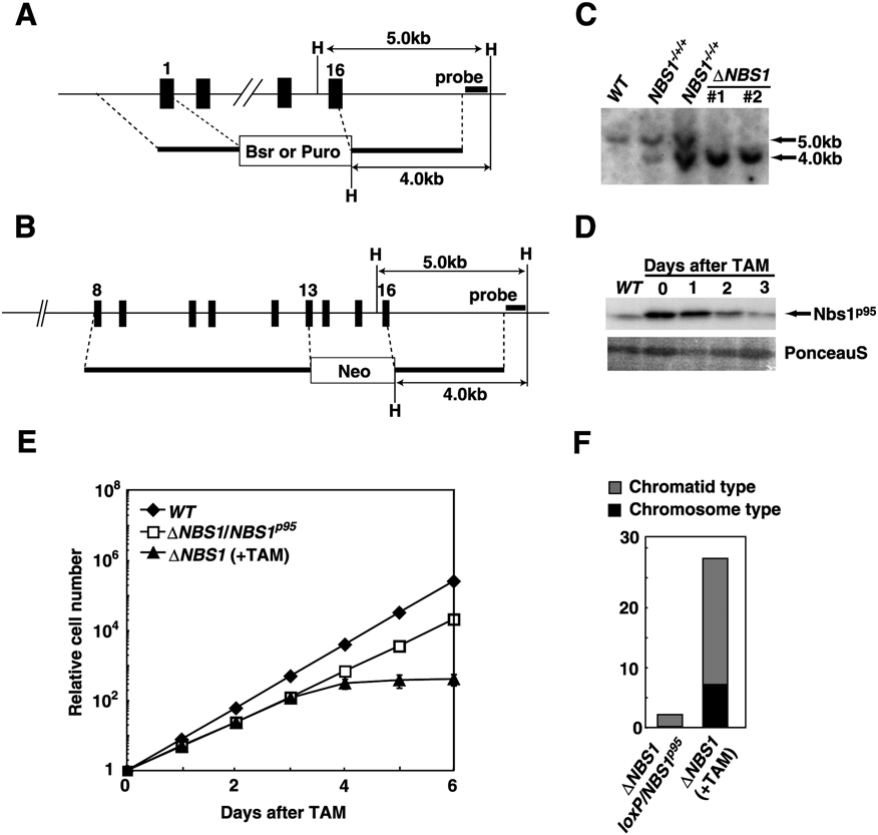
*NBS1*-disrupted mutants are lethal to cells. (A, B) The two *NBS1*-disrupting constructs for *NBS1 Δ1–16* (A) and *NBS1 Δ13–16* (B) are shown in comparison with the relevant *NBS1* genomic sequences (top). Solid boxes indicate the position of exons. Only disrupted exons are indicated. Relevant *Hin*dIII sites and the position of the probe used in the Southern-blot analysis (C) are indicated. (C) *NBS1* gene-targeting was confirmed by Southern-blot analysis of *Hin*dIII-digested genomic DNA. Position of hybridizing fragments of the *WT* and targeted loci are indicated. (D) Depletion of Nbs1 protein in *ΔNBS* cells during tamoxifen treatment was analyzed by western blotting analysis. ∼50 kD Ponceau S-stained bands was used as a loading control. (E) Growth curves corresponding to the indicated cell cultures. Experiments were done at least three times. +TAM represents continuous exposure to tamoxifen. Error bars represent standard deviation (SD). (F) Accumulation of chromosomal breaks in Nbs1-deficient mitotic cells. The *ΔNBS loxP-NBS1^p95^* cells were exposed to tamoxifen for 4 days to excise the *loxP-NBS1^p95^* transgene. Fifty mitotic cells were analyzed in each case.

The chicken *NBS1* gene is located on chromosome 2, which is trisomic in DT40 cells. To completely inactivate the *NBS1* gene, we generated deletion constructs containing different marker genes, a procedure designed to remove the entire reading frame of the *NBS1* gene, including all 16 exons (∼30 kb) (Figure 1A). These targeting plasmids were sequentially transfected into *wild-type* (*WT*) DT40 cells, and the *NBS1^−/−/+^* cells were isolated. To generate conditional *NBS1*-disrupted cells, we employed Cre-recombinase-mediated deletion of a chicken *NBS1* transgene. *NBS1^−/−/+^* cells were transfected with the transgene containing the *WT NBS1* cDNA flanked by *loxP* sites on both sides (the *loxP-NBS1^p95^* transgene) together with a Cre-ER expression vector [46]. The resulting *NBS1^−/−/+^*/*loxP-NBS1^p95^* clones were transfected with targeting constructs to disrupt exons 1–16 or exons 13–16, which encodes the Mre11-binding domain of the third *NBS1* allele (Figure 1B). We were only able to obtain targeted integration with the latter construct, because Nbs1 overproduction from the *loxP-NBS1^p95^* transgene substantially reduced gene-targeting efficiency. The genotype of the *NBS1^−/−/Δ13–16^*/*loxP-NBS1^p95^* (hereafter *ΔNBS1*/*loxP-NBS1^p95^*) clones was confirmed by Southern-blot analysis of *Hin*dIII-digested genomic DNA for the disappearance of a *WT* 5 kb band (Figure 1C). Western-blot analysis showed that *ΔNBS1*/*loxP-NBS1^p95^* cells expressed levels of Nbs1^p95^ that were about 50 fold higher than the *WT* cells (Figure 1D). *ΔNBS1*/*loxP-NBS1^p95^* cells tended to grow more slowly than did *WT* cells (Figure 1E), a phenotype that may be attributed to the overexpressed *NBS1^p95^*.

To investigate whether Nbs1^p95^ is required for cellular proliferation, *ΔNBS1*/*loxP-NBS1^p95^* cells were treated with tamoxifen to activate the Cre recombinase, resulting in the deletion of the *loxP-NBS1^p95^* transgene. *ΔNBS1*/*loxP-NBS1^p95^* cells ceased proliferating four days after the addition of tamoxifen (Figure 1E), with substantial numbers of dead cells (data not shown). These observations indicate that *NBS1* is required for cellular proliferation, as previously reported [47]. To investigate the cause of the cell death, we scored spontaneous chromosomal aberrations when the cells were dying. The tamoxifen-treated *ΔNBS1*/*loxP-NBS1^p95^* cells indeed exhibited extensive spontaneous chromosomal breaks (Figure 1F), as did Mre11 deficient cells [13], indicating an essential role for Nbs1 in repairing lethal double-strand breaks.

We also made conditional Rad50-depleted cells and found that they too exhibited an increase in the level of chromosomal breaks before cell death (Figure S1). Thus, a loss of Mre11, Rad50 and Nbs1 has a very similar effect on the maintenance of chromosomal DNA in cycling cells, suggesting that the three molecules form a functional unit, as do the yeast ortholog proteins [5].

**Figure 2:**
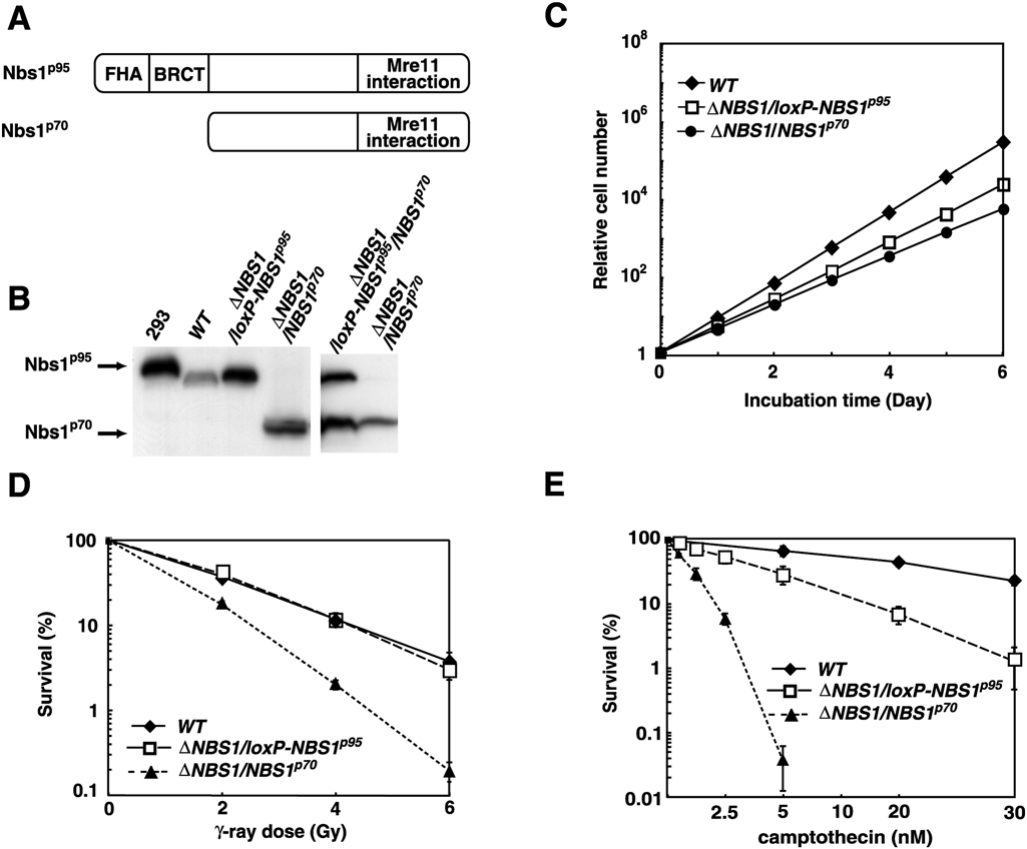
C-terminal p70 Nbs1 protein can rescue the lethality of *NBS1* deleted cells. (A) Schematic representation of Nbs1 proteins. Nbs1^p95^ represents a *WT* protein. (B) Western-blot analysis of Nbs1. An anti-Nbs1 antibody that recognizes the C-terminal region of the Nbs1 protein was used. 293: HEK293 cell extract was used as a control. (C) Growth curves corresponding to the indicated cell cultures. Experiments were done at least three times. Error bars (SD) are too small to show. (D, E) Sensitivity of *WT*, *ΔNBS1/loxP-NBS1^p95^* and *ΔNBS1*/*NBS1^p70^* cells to γ-rays (D) and a topoisomerase I inhibitor, camptothecin (E). The fractions of surviving colonies after treatment of cells compared with untreated controls of the same genotype are shown on the y-axis as a logarithmic scale. The doses of γ-rays and camptothecin are displayed on a linear scale on the x-axis of each graph. The data shown are representative results from three separate experiments. Error bars represent SD.

We wanted to test whether or not expression of Nbs1^p70^ could rescue the cells from cell death. To this end, we complemented *ΔNBS1*/*loxP-NBS1^p95^* cells with an *NBS1^p70^* transgene and generated *ΔNBS1*/*loxP-NBS1^p95^*/*NBS1^p70^* clones. The Nbs1^p70^ protein contains an Mre11-binding site, but lacks both the FHA and BRCT domains (Figure 2A) [5]. Western-blot analysis verified the Nbs1^p70^ expression, which was about 30 times higher than the expression of endogenous Nbs1 (Figure 2B). To remove the *loxP-NBS1^p95^* transgene, *ΔNBS1*/*loxP-NBS1^p95^*/*NBS1^p70^* cells were exposed to tamoxifen for three days, and isolated clones were examined for the expression of the Nbs1 protein. All surviving colonies expressed Nbs1^p70^, but not *WT* Nbs1^p95^, showing that their genotype is *ΔNBS1*/*NBS1^p70^* (Figure 2B). The resulting clones proliferated with slightly slower kinetics than did the *ΔNBS1*/*loxP-NBS1^p95^* cells (Figure 2C). We therefore conclude that Nbs1^p70^ is sufficient to rescue *NBS1*-deficient cells. This conclusion implies that the viability of previously described Nbs1-deficient DT40 cells might be attributable to the leaky expression of an N-terminally truncated protein [48].

### Reduction in the Rate of Ig Gene Conversion in Nbs1^p70^-Expressing Cells

Two representative *ΔNBS1*/*NBS1^p70^* clones were further studied for their HR capability by measuring their gene-targeting frequency and sensitivity to DNA-damaging agents. Table 1 shows the ratio of targeted-to-random integration events at two loci. No gene-targeting events were detectable in the *ΔNBS1*/*NBS1^p70^* clones. We next measured cellular sensitivity to ionizing radiation and camptothecin, a DNA-topoisomerase-I inhibitor [49]. Ionizing-radiation-induced DSBs are repaired by the two major DSB repair pathways, HR and nonhomologous end-joining [50], whereas camptothecin-induced DSBs are repaired exclusively by HR [51]–[53]. Compared with *WT* cells, the *ΔNBS1*/*NBS1^p70^* cells showed a significant increase in damage sensitivity, particularly to camptothecin (Figures 2D and E). This is consistent with previous reports showing that Nbs1 promotes HR-mediated DSB repair [47],[48].

**Table 1:** Targeted integration frequencies in the indicated loci.

Genotype	Targeted integration frequencies	
	Targeted locus	
	*OVALBUMIN*	*HPRT*
*WT*	40/46 (87.0%)	12/34 (35.3%)
*ΔNBS1/loxP-NBS1^p95^*	8/27 (29.6%)	3/37 (8.1%)
*ΔNBS1/NBS1^p70^*	0/46 (0%)	0/46 (0%)

*WT*, *ΔNBS1/loxP-NBS1^p95^* and *ΔNBS1/NBS1^p70^* cells were transfected with targeting constructs of the indicated loci. The data shown are the number of targeted events at each locus divided by the number of drug-resistant clones analyzed. The percent frequency is in parentheses.

**Figure 3:**
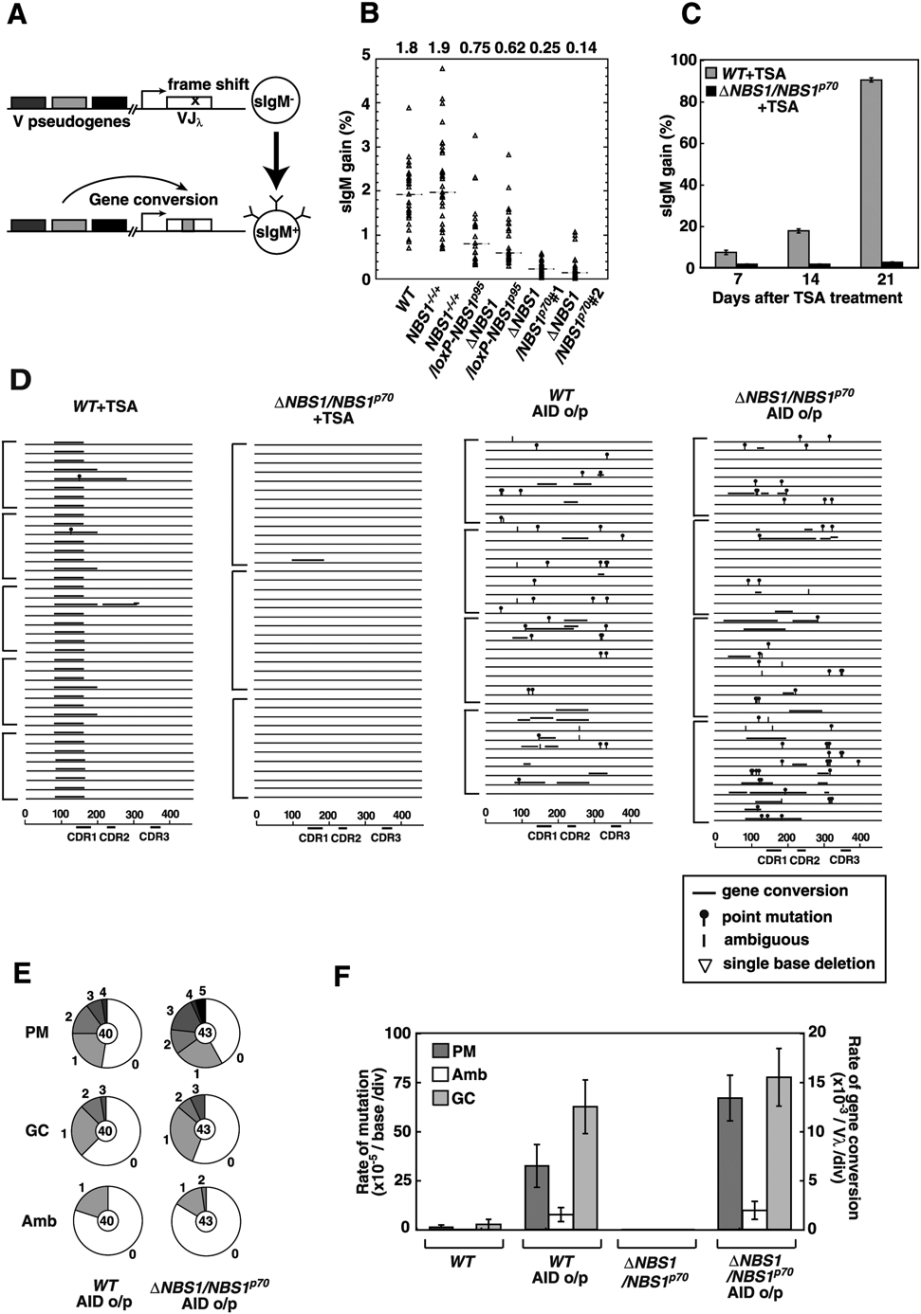
Rate of Ig gene conversion reduced in *ΔNBS1*/*NBS1^p70^* cells. (A) Schematic outline of the Ig gene conversion fluctuation assay in DT40 cells. The cross in the rearranged VJ_λ_ shows the frame-shift mutation that inhibits surface IgM expression. (B) The abundance of sIgM-gain variants was determined in 36 parallel cultures derived from single sIgM-negative parental cells after clonal expansion (3 weeks). Median percentages are noted above each data set and indicated by dashed lines. (C) Kinetics of sIgM-gain variants as a percentage of total cells during tricostatin A (TSA) treatment. Error bars represent the SD of three independent cultures. (D) Ig gene conversion and single-base substitution events in tricostatinA-treated *WT* and tricostatinA-treated *ΔNBS1*/*NBS1^p70^*, AID overexpressing *WT* and AID overexpressing *ΔNBS1*/*NBS1^p70^* cells. Each horizontal line represents the rearranged V_λ_ (450 bp), with mutations classified, as described in the text, as non-templated base substitution (lollipop shape), long-tract gene conversion (horizontal bar above line), single nucleotide substitutions that could be a result of point mutation or gene conversion (vertical bar) and single-base deletion (open triangle), determined as previously described [20]. Clones were expanded for 2 weeks for AID overexpression and 4 weeks for TSA treatment. More than three clones were analyzed for each data set. Nucleotide sequence data included in one square bracket are derived from individual cell clones. (E) Proportion of non-templated single-base substitution (PM), long-tract gene conversion (GC) and mutations of ambiguous origin (Amb). Segment sizes are proportional to the number of sequences carrying the number of mutations indicated around the outside of the pie chart at individual V_λ_ segments. The total number of V_λ_ sequences analyzed is indicated in the center of the chart. (F) A graphical view showing the rates of non-templated single-base substitution (PM), point mutations of ambiguous origin (Amb) and Ig gene conversion (GC) in *WT* cells, AID overexpressing *WT* cells, *ΔNBS1*/*NBS1^p70^* cells and AID overexpressing *ΔNBS1*/*NBS1^p70^* cells. Clones were expanded for 2 weeks. More than two clones were analyzed for each data set.

The rate of Ig gene conversion was assessed by measuring the re-expression of surface immunoglobulin M (sIgM) in DT40 clones that carry a defined frameshift mutation in the light-chain V_λ_ gene [21]. Since the frameshift is eliminated by superimposed Ig gene conversion, leading to the production of Igλ, the rate of Ig gene conversion can be evaluated by measuring the kinetics of sIgM gain (Figure 3A). Thirty subclones from each genotype were analyzed for sIgM-gain after 3 weeks of clonal expansion [33],[54]. The median value of the fraction of sIgM^+^ cells was 1.84% for *WT*, 1.91% for *NBS1^−/−/+^* and 0.75% for *NBS1^−/−/+^*/*loxP-NBS1^p95^* cells (Figure 3B). The reduced Ig gene conversion rate in *NBS1^−/−/+^*/*loxP-NBS1^p95^* cells may result from the toxic effect of the overproduced Nbs1^p95^ protein. Two *ΔNBS1*/*NBS1^p70^* clones displayed a significant decrease in gene conversion, with only 0.1–0.2% of subclones gaining sIgM, a level close to the background of the flow-cytometric analysis. To accurately evaluate the Ig gene conversion rate, we exposed populations of cells to trichostatin A, a histone-deacetylase inhibitor that increases the Ig gene conversion rate ∼50 fold [55],[56]. Following culture for 3 weeks in trichostatin A, the sIgM gain was only elevated to 2.15% in the *ΔNBS1*/*NBS1^p70^* cells, while the *WT* cells exhibited an increase from 1.84 to over 90% (Figure 3C). This suggests that the intact MRN complex might promote Ig gene conversion, as reported previously [57]. Alternatively, the accuracy of Ig gene conversion in the *ΔNBS1*/*NBS1^p70^* cells might be reduced, leading to a decrease in the re-expression of sIgM.

To examine the accuracy of Ig gene conversion, we determined the VJ_λ_-nucleotide sequences from unsorted cells treated with trichostatin A for 4 weeks (Figure 3D). In trichostatin-A-treated unsorted *WT* cells, at least 42 Ig gene conversion events were detected among the 40 analyzed V_λ_ segments (1.3×10^−2^ events per V_λ_ per division). In contrast, the number of Ig gene conversion tracts was only one in 40 analyzed V_λ_ (3×10^−4^ events per V_λ_ per division) in *ΔNBS1*/*NBS1^p70^* cells. This 42-fold difference is comparable to the difference observed in the sIgM-gain assay (Figure 3C). Ig V sequence analysis showed that the accuracy of these events is unaffected, as neither aberrant recombination nor accumulation of point mutations was found in *ΔNBS1*/*NBS1^p70^* cells. To characterize the nature of Ig gene conversion, we also analyzed the VJ_λ_ nucleotide sequences of sorted sIgM^+^ revertants from *ΔNBS1*/*NBS1^p70^* trichostatin-A-untreated cell populations. The frame-shift mutation in Ig V_λ_ [21] was indeed eliminated by superimposed gene conversion in all 40 analyzed fragments derived from *ΔNBS1*/*NBS1^p70^* cells (data not shown). Furthermore, we found no change in the pattern of gene conversion, such as length of gene-conversion tracts (84 nucleotides on average for both *ΔNBS1*/*NBS1^p70^* and *WT* [56]) or usage of pseudo-V donor segments, and no aberrant recombination (data not shown). Thus, although the defective Nbs1 function reduces the rate of Ig gene conversion, it compromises neither its accuracy nor donor gene preference.

### Effect of Ectopic Expression of AID on Defective IG Gene Conversion in Nbs1-Deficient Cells

To analyze Ig V hypermutation in *ΔNBS1*/*NBS1^p70^* cells, we increased the level of AID expression by introducing an AID transgene into DT40 cells through retroviral infection [31],[58]. We assessed Ig V diversification by determining the nucleotide sequence of Ig V_λ_ in unsorted cells at 14 days post-infection (Figure 3D). *WT* and *ΔNBS1*/*NBS1^p70^* cells exhibited similar levels of non-templated hypermutation: about 5.0×10^−4^ per nucleotide per division (Figure 3F). Thus, a defect in Nbs1 does not affect Ig V hypermutation.

AID overexpression increased the rate of Ig gene conversion from 5.2×10^−4^ to 1.3×10^−2^ per V_λ_ per division in 40 analyzed V_λ_ in *WT* cells (Figures 3E and F). Surprisingly, the frequency of Ig gene conversion in *ΔNBS1*/*NBS1^p70^* cells reached the level of the *WT* cells, i.e., 1.6×10^−2^ per V_λ_ per division in 40 analyzed V_λ_ sequences. Thus, the frequency of gene conversion was increased 25 fold in *WT* cells and 307 fold in *ΔNBS1*/*NBS1^p70^* cells by the ectopic expression of AID. No aberrant recombination events were observed. We conclude that a defect in Ig gene conversion in *ΔNBS1*/*NBS1^p70^* cells is completely normalized by the ectopic expression of AID. This observation suggests two scenarios, described as follows: DSBs might initiate Ig gene conversion in a manner similar to the way in which AID-dependent DSBs trigger Ig-class switch recombination (reviewed in [59]). Thus, higher levels of AID expression may result in multiple deamination events on both strands, with the ensuing incisions more likely to generate DSBs carrying the 3′ tails even in the absence of the intact MRN complex. Alternatively, Ig gene conversion might be initiated by single-strand gaps. In the latter model, the formation of multiple abasic sites and incisions in one strand results in the generation of recombinogenic single-strand gaps, after which Nbs1^p70^ is no longer required for the processing of single-strand lesions to stimulate Ig gene conversion.

### No Involvement of DSBs in AID-Induced Ig Gene Conversion

**Figure 4:**
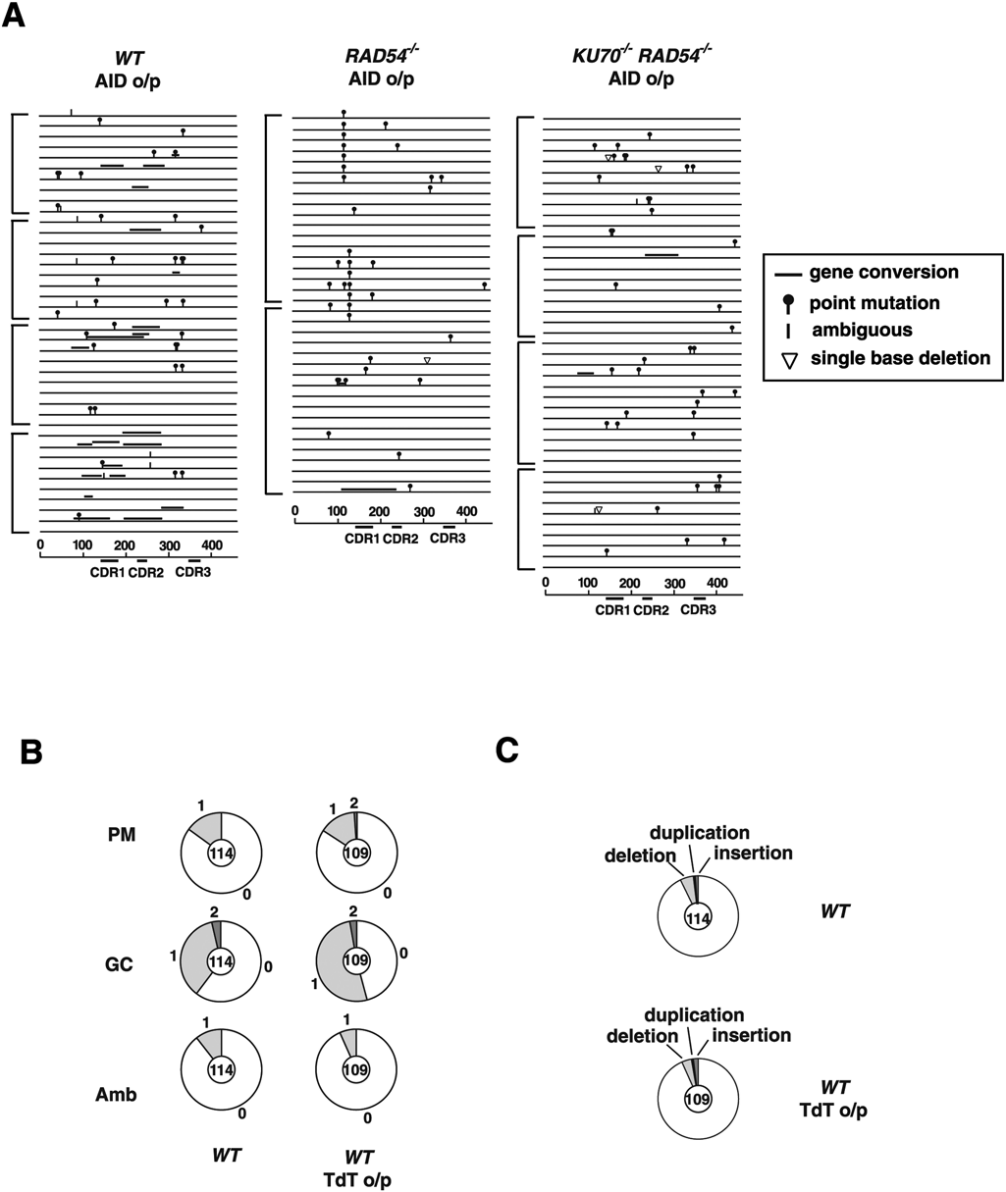
Double-strand breaks may not be associated with Ig gene conversion. (A) Ig V mutation events in AID overexpressing *WT*, *RAD54^−/−^* and *KU70^−/−^RAD54^−/−^* cells. Each horizontal line represents the rearranged V_λ_ (450 bp) with mutations classified as described in Figure 3D. Clones were expanded for 2 weeks. More than two clones were analyzed for each data set. Nucleotide sequence data included in one square bracket are derived from individual cell clones. (B) (C) Immunoglobulin diversification in terminal deoxytransferase- (TdT) expressing DT40 cells. (B) Proportion of sequences carrying the indicated number of non-templated point mutations (PM), ambiguous mutations (Amb) and gene conversions (GC), determined as previously described [20]. The total number of sequences analyzed is indicated in the center of the pie chart. Data are derived from four independent clones. Control data for the TdT-expression experiment (*WT* with no TdT expression) were previously published [20] and are included here for comparison. (C) Proportion of sequences containing a deletion, duplication or insertion.

There are two major DSB repair pathways: HR and nonhomologous end-joining (NHEJ). Two studies previously reported the negative effect of NHEJ on Ig gene conversion [60],[61], which suggests that DSBs are an intermediate in Ig gene conversion. However, the IgV sequence from unsorted populations show only a two-fold increase [60] or no increase [61] in the rate of Ig gene conversion in NHEJ deficient clones in comparison with WT cells. Furthermore, another study [20] and our own work did not reproduce their data (data not shown). In general, it is difficult to draw a conclusion from at best a two-fold difference due to possible clonal variations in DT40 cells. To determine the involvement of DSBs in Ig gene conversion more accurately, we performed two experiments: 1) Detection of deletions within V_λ_ in *RAD54^−/−^* and *KU70^−/−^RAD54^−/−^* clones [50] (Figure 4A), and 2) terminal deoxytransferase (TdT) expression (Figure 4B and C).

In the first experiment, the effect of Ku70 depletion on Ig V diversification was investigated in the *RAD54^−/−^* background, where HR is not completed despite the accumulation of Rad51 at sites with DNA damage [62],[63]. Since the loss of Rad54 is substantially suppressed by NHEJ in the repair of x-ray-induced DSBs [50], we assumed that if Ig gene conversion is initiated by DSBs, a majority of such breaks would eventually be repaired by NHEJ in *RAD54^−/−^* cells, as are x-ray-induced DSBs. Thus, the additional inactivation of Ku70 in *RAD54^−/−^* cells would result in the deletion of V_λ_ sequences, as illustrated by the extensive deletion of the V(D)J coding joint in NHEJ-defective B precursors [64]. To detect deletion of Ig V_λ_, we determined the nucleotide sequences of V_λ_ in AID overexpressing *WT*, *RAD54^−/−^* and *KU70^−/−^RAD54^−/−^* cells (Figure 4A). The *RAD54^−/−^* and *KU70^−/−^RAD54^−/−^* cells exhibited only one (6.6×10^−4^ per V_λ_ per division in 36 analyzed V_λ_ sequences) and three (1.7×10^−3^ per V_λ_ per division in 43 analyzed V_λ_ sequences) single-nucleotide deletion events, respectively. There were no longer deletions. Thus, unlike the repair of x-ray-induced DSBs, this result does not support the idea that unrepaired AID-induced damage at the V_λ_ segment of *RAD54^−/−^* cells is subject to NHEJ-mediated DSB repair.

In the second experiment, we overexpressed TdT, which added nucleotides at DSBs in a template-independent manner during V(D)J-joining [65],[66]. TdT has been shown to access the Ig locus when expressed in a human cell line that undergoes constitutive Ig somatic mutation *in vivo* [67]. If DSBs are a frequent trigger for Ig gene conversion, TdT-mediated nucleotide additions should be readily demonstrated at Ig V_λ_ in DT40 cells expressing TdT. We therefore transfected a TdT expression plasmid into *WT* DT40 cells and performed an Ig V_λ_ sequence analysis. The TdT overproduction affected neither point mutation nor Ig gene conversion frequency (Figure 4B). In contrast to the effect seen in hypermutating Ramos cells [67], we could not detect any difference in insertion frequency between *WT* cells with or without TdT overproduction (Figure 4C). Furthermore, all the insertions were of a single base pair, with the exception of one sequence where a deletion of 19 base pairs was associated with the insertion of CCC, which could not be accounted for by a pseudogene donor (ACAACGT**CCC**..19 bp del…GACAACC). This is the only example within the analyzed 109 sequences that may reflect the activity of TdT. The absence of additional nucleotides at Ig V_λ_ indicates that DSBs are not intimately associated with Ig gene conversion.

In summary, these data support the hypothesis that the initiating lesions for Ig gene conversion are predominantly single-strand gaps rather than DSBs. Hence, AID overexpression that normalizes the impaired Ig gene conversion of *ΔNBS1*/*NBS1^p70^* cells (Figure 3D) possibly does so as a consequence of the formation of multiple incisions in one strand, which promotes the generation of recombinogenic single-strand gaps even in the absence of the intact MRN complex. This hypothesis is also supported by a previous biochemical study, which demonstrates that AID processively deaminates C residues on a single-strand DNA [68].

### SbcB, a Nuclease that Acts on a Single-Strand Gap, Induces Gene Conversions in *nbs1*-Deficient Cells

**Figure 5:**
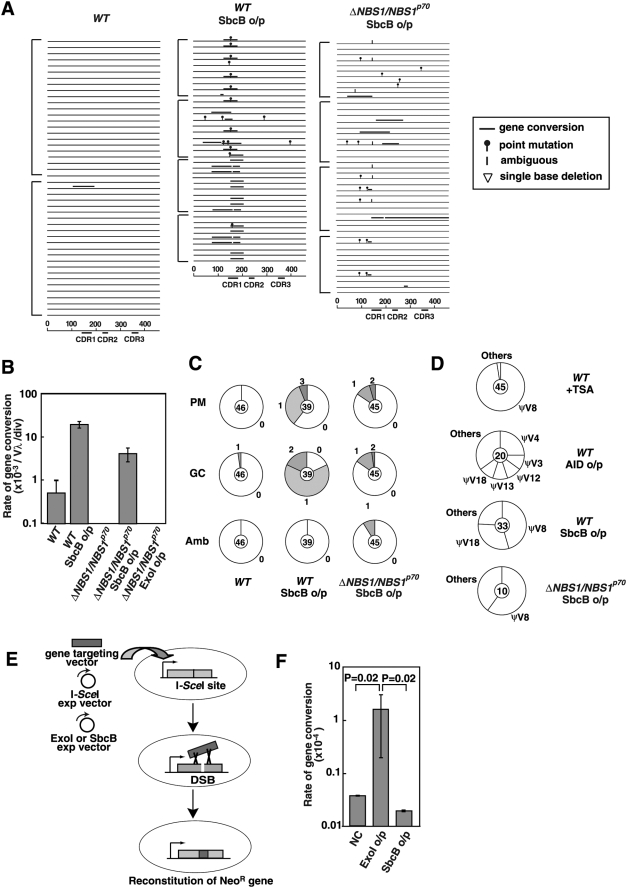
SbcB restores the Ig gene conversions of nbs1-deficient cells. (A) Ig V diversification of *WT* cells, *WT* cells expressing SbcB, and *ΔNBS1*/*NBS1^p70^* cells expressing SbcB. Each horizontal line represents the rearranged V_λ_ (450 bp) with mutations classified as described in Figure 3D. Clones were expanded for 2 weeks. More than two clones were analyzed in each data set. Nucleotide sequence data included in one square bracket are derived from individual cell clones. (B) Comparison of Ig gene conversion rates in *WT* DT40 cells expressing SbcB, *ΔNBS1*/*NBS1^p70^* cells and *ΔNBS1*/*NBS1^p70^* cells carrying SbcB or the Exo1 transgene. Note that no gene conversion event was detectable in *ΔNBS1*/*NBS1^p70^* cells or in those expressing the chicken Exo1 transgene. (C) Proportion of non-templated single-base substitution (PM), long-tract gene conversion (GC) and mutations of ambiguous origin (Amb). Data are shown as in Figure 3E. (D) Preference of pseudo- (Ψ) V-gene usage as a donor for Ig gene conversion events in unsorted tricostatinA- (TSA) treated *WT*, *WT* cells overexpressing AID, *WT* cells expressing SbcB and *ΔNBS1*/*NBS1^p70^* cells expressing SbcB. The total number of gene conversion events is shown in the central circles. (E) Experimental method of measuring the frequency of gene-targeting by counting neomycin-resistant colonies. The expression vector of I-*Sce*I and the 3′*neo* gene-targeting DNA (dark gray box) are introduced, together with either the negative control vector, the SbcB or the chicken Exo1 expression plasmid, into cells carrying *S2neo* in the *Ovalbumin* locus. Successful gene-targeting would reconstitute a functional *neo* ^R^ gene. (F) The frequency of gene-targeting is shown as the number of G418-resistant colonies derived from 10^7^ transfected cells [44]. The experiments were done more than five times. NC indicates transfection with the negative-control vector. Note that no gene-targeting event was detectable without I-*Sce*I expression. Error bars represent SD.

If Ig gene conversion is triggered by single-strand lesions, then the MRN complex is likely to contribute to Ig gene conversion, possibly by converting small single-strand lesions to larger, more recombinogenic gaps. To test this hypothesis, we attempted to normalize the impaired Ig gene conversion of the Nbs1-deficient cells by overproducing nucleases whose activity is precisely characterized. These nucleases included Exo1 [36]–[40] and SbcB [41]–[43]. Using a retroviral vector, we introduced individual nuclease transgenes into DT40 cells and established overproducing clones. We cultured individual clones for 2 weeks and determined the nucleotide sequences of the V_λ_ segment. Remarkably, SbcB dramatically increased the rate of Ig gene conversion in *ΔNBS1*/*NBS1^p70^* (Figure 5A–C). Unexpectedly, this increase was not observed in *ΔNBS1*/*NBS1^p70^* cells overexpressing chicken Exo1, presumably because this exonuclease can work only in a physiological context such as during mismatch repair in the chicken cell line. The frequency of Ig gene conversion in SbcB overproducing Nbs1-deficient cells reached 4.2×10^−3^ per V_λ_ per division in analyzed 45 V_λ_ sequences, a level higher than the gene-conversion frequency of the *WT* cells (Figure 5B). Ectopic SbcB expression did not significantly change the position (compare Figures 3D and 5A) or pseudo-V usage (Figure 5D) of the Ig gene conversion. In contrast, the nature of the Ig gene conversion was distinctly different between trichostatin-A-treated *WT* cells and those overproducing AID (Figure 3D and 5A). Presumably, this is because, according to a previous biochemical study [68], overproduced AID can deaminate even “cold” spots at Ig V, thereby initiating HR from a wider range of nucleotide sequences than does the endogenous AID of DT40 cells. Thus, it is likely that SbcB promotes Ig gene conversion in the same physiological manner as does the MRN complex. SbcB has the 3′ to 5′ exonuclease activity specific for single-stranded DNA *in vitro* [41]–[43], and can thereby expand single-strand gaps to stimulate HR *in vivo*. Hence, we conclude that the MRN complex contributes to Ig gene conversion in a similar manner by increasing the size of single-strand gaps.

To test whether overproduced SbcB affects HR-dependent repair of DSBs *in vivo*, we measured the effect of SbcB overproduction on DSB repair. To this end, we measured I-*Sce*1-induced gene-targeting [44]. We inserted the *S2neo* fragment carrying the I-*Sce*I recognition site [69] into the *OVALBUMIN* locus of DT40 cells, subsequently transfecting the *3′neo* fragment [69] (gene-targeting vector in Figure 5E) together with an I-*Sce*I expression plasmid. Since gene-targeting of *3′neo* into *S2neo* leads to the restoration of the *WT* neomycin-resistance (*neo^R^*) gene, the efficiency of gene-targeting events can be analyzed by measuring the frequency of *neo^R^* colonies. As previously observed [45], the co-transfection of the I-*Sce*I-expression plasmid increases the gene-targeting frequency of *3′neo* by more than three orders of magnitude. To test whether SbcB affects DSB-induced gene-targeting, we measured gene-targeting frequency following transfection of both the *3′neo* gene-targeting fragment and the I-*Sce*I-expression plasmids, along with either a nuclease-expression-plasmid (SbcB or the chicken Exo1-expression plasmids) or a negative control vector into *WT* DT40 cells. The ectopic expression of SbcB had no impact on DSB-induced gene-targeting (Figure 5F). In contrast, overproduction of chicken Exo1 increased the frequency of gene-targeting events more than 10 fold. This observation argues against the involvement of overproduced SbcB in DSB repair.

## Discussion

We show in this study that DT40 cells deficient in the individual components of the MRN complex exhibit similar phenotypes, including extensive chromosomal breaks prior to cell death. This observation suggests that Nbs1 participates in HR as part of the MRN complex, as does the MRX complex in yeast. As expected, the lethality of Nbs1-deficient cells was rescued by the expression of the Nbs1^p70^ N-terminal-truncated protein. *ΔNBS1*/*NBS1^p70^* cells showed a significant decrease in the rate of Ig gene conversion. In the following subsections we present evidence that suggests that Ig gene conversion may be initiated by AID-induced single-strand lesions and that the MRN complex contributes to Ig gene conversion presumably by processing these single-strand lesions to generate recombinogenic gaps.

### Ig Gene Conversion Events Are Initiated by Single-Strand Lesions but not by DSBs

**Figure 6:**
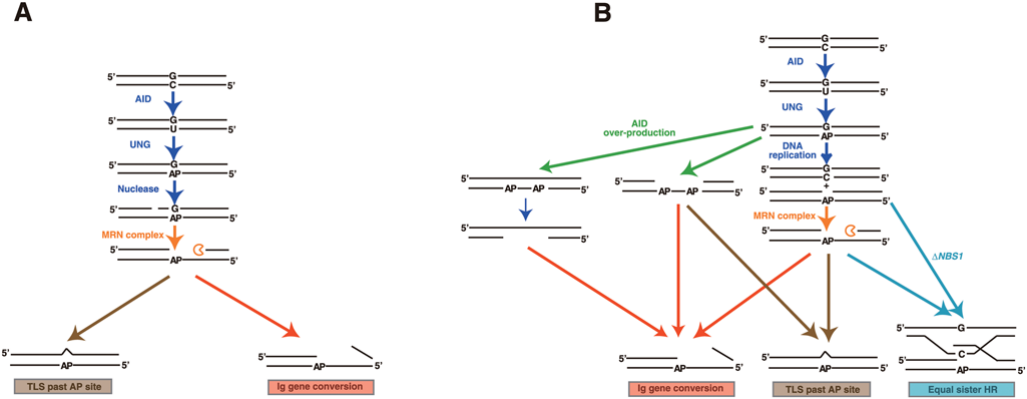
Model for single-strand-lesion-induced Ig gene conversion. At the Ig V, AID (activation induced deaminase) induces an abasic (AP) site, which is required for both Ig gene conversion and translesion-DNA-synthesis-mediated hypermutation at the dC∶dG pair. Single-strand breaks are generated at the opposite site of an abasic site either by a putative endonuclease (A) or as a consequence of the block of replication forks (B). In both cases, the MRN complex expands the single-strand lesions to trigger HR. If Rad51 does not accumulate effectively, translesion DNA synthesis past abasic sites leads to hypermutation at the dC∶dG pair. In model B, equal sister recombination may dominate in a *ΔNBS1*-deficient background, resulting in suppression of both Ig gene conversion and hypermutation. Following overexpression of AID in *ΔNBS1* cells, extensive processing of single-strand lesions by the MRN complex becomes dispensable for Ig gene conversion (A). AID overproduction leads to the formation of single-strand gaps due to replication blocks at multiple abasic sites (B). In Nbs1-deficient cells, single-strand lesions preferentially stimulate equal sister recombination, and thus do not effectively induce Ig gene conversion or translesion DNA synthesis.

Two mechanisms could underlie the AID-dependent initiation of Ig gene conversion. The first assumes that AID-dependent single-strand lesions are converted to DSBs (possibly by blocking replication in one of the two sister chromatids), which stimulate Ig gene conversion. The second states that AID-dependent single-strand lesions directly trigger Ig gene conversion. The first scenario is unlikely for five reasons. First, in *brca1*, *brca2* and *rad51-paralog* DT40 mutants, which are defective in the accumulation of Rad51 at sites of DNA damage, inefficient repair of AID-induced lesions activates TLS associated with hypermutation at dC∶dG basepairs [20],[32],[33]. Thus, the AID-induced substrate for HR is also likely to be the substrate for TLS-dependent Ig V hypermutation. Since effective TLS requires that there is no cleavage of the abasic-site-containing strand, it seems therefore plausibe that unfilled gaps directly stimulate Ig gene conversion in HR-proficient cells. (Figure 6). Second, if AID directly causes DSBs in Ig V_λ_, such breaks would likely be repaired primarily by NHEJ in HR-deficient cells. Although it has been shown that AID-mediated DSBs trigger Ig-class switch recombination, which is partially dependent on NHEJ-mediated DSB repair [70], we did not obtain evidence for the involvement of NHEJ in Ig gene conversion, even in *RAD54^−/−^* cells (Figure 4A), where a late step of HR is compromised [62],[63]. This observation conflicts with the critical role NHEJ plays in the repair of X-ray-induced DSBs, as evidenced by the significant increase in sensitivity to x-rays in *KU70^−/−^RAD54^−/−^* cells compared with *RAD54^−/−^* cells [50]. Third, overexpression of terminal deoxytransferase failed to add extra-nucleotide sequences at the Ig V_λ_ of DT40 cells (Figure 4B and C). This observation argues against the significant association of Ig gene converstion with DSBs, because N nucleotides are inserted at the DSBs, as observed in DSB-induced V(D)J recombination [65],[66]. Fourth, although chicken Exo1 overproduction significantly increased the frequency of DSB-induced HR (Figure 5F), as observed in yeast [38],[39], the overproduction of SbcB did not enhance DSB-induced gene-targeting. However, SbcB reversed the defective Ig gene conversion in the Nbs1-deficient DT40 cells. Moreover, it is believed that SbcB suppresses DSB-induced HR, because its 3′ to 5′ exonuclease activity may remove the 3′ protruded tails from DSBs (reviewed in [3]). Collectively, these data suggest that DSBs do not play a major role in triggering Ig gene conversion, and that it is more likely that single-strand gaps formed by the sequential action of AID, UNG and the MRN complex directly stimulate Ig gene conversion.

At one time, models for both DSB- and nick-initiated HR were proposed [71],[72] (reviewed in [1]). The finding of DSBs during meiosis, as well as the development of the restriction-enzyme-induced HR model, established the DSB as the main initiator of HR [73],[74]. However, accumulating evidence indicates that single-strand lesions are indeed responsible for the initiation of HR in both RecFOR-dependent HR in *E. coli* and in mutant V(D)J recombinase-induced HR in episomal plasmids [2],[75]. Adding to this evidence, our study indicates that Ig gene conversion is a form of HR that is directly stimulated by single-strand lesions on chromosomal DNA in higher eukaryotic cells. The question remains as to whether or not single-strand gap-induced HR effectively contributes to the release of the replication block in the absence of accompanying DSBs.

### Role of the MRN Complex in Ig Gene Conversion

The notion that single-strand lesions directly stimulate Ig gene conversion indicates that, like SbcB, the MRN complex may promote HR by converting single-strand breaks to more recombinogenic substrates such as single-strand gaps. In fact, according to the nick-initiating HR model, the initial nick is expanded into a single-strand gap to trigger HR [72]. Moreover, the presence of such activity is suggested by the biochemical study of CtIP, a protein that physically interacts with the MRN complex [8],[10]. On the other hand, Larson et al. indicate that the MRN complex incises a strand near an abasic site [76]. However, if this activity plays a dominant role in the initiation of Ig gene conversion, one cannot explain why the subsequent defect in the accumulation of Rad51 at the incision in the *rad51 paralog* and *brca* mutant shift Ig V diversification from HR- to TLS-mediated hypermutation [20],[32],[33]. Nonetheless, it is possible that the incision activity accounts for a fraction of Ig gene conversion. A defect in this incision activity might be substituted by AID overexpression, as it could introduce multiple AP sites, which makes less effective AP endonuclease compensate for the defective incision activity of the mutant Mre11 complex in *ΔNBS1*/*NBS1^p70^* cells.

Figure 6 presents two models for the participation of the MRN complex in Ig gene diversification. In both models, AID-mediated catalysis and subsequent hydrolysis of uracil lead to the formation of abasic sites. The first model assumes an endonuclease that can cleave the opposite strand of the abasic-site-containing strand (Figure 6A), while the second model hypothesizes single-strand gap formation as a result of stalled replication (Figure 6B). The MRN complex facilitates HR by increasing the length of gaps in both models. Quick and copious recruitment of Rad51 at DNA lesions triggers Ig gene conversion, whilst poor recruitment leads to translesion DNA synthesis past abasic sites by error-prone polymerases. In the second model, it is still unclear why, despite the 10% sequence divergence between pseudo-V donor and V(D)J recipient fragments, competition between equal sister-chromatid HR and Ig gene conversion (Figure 6B) does not fully inhibit homologous recombination in the latter [24]. Presumably, extensive processing of single-strand lesions by the MRN complex and SbcB allows for homologous recombination, whilst impaired processing inhibits both TLS and Ig gene conversion (Figure 6B). The overproduction of AID might form gaps between two adjacent abasic sites on one strand, thereby suppressing the defective processing of single-strand lesions in *ΔNBS1*/*NBS1^p70^* cells (Figure 6B). Additionally, the MRN complex contributes to Ig gene conversions through its incision activity [76], and its defect in *ΔNBS1*/*NBS1^p70^* cells is rescued by the formation of multiple AP sites in AID overexpressing cells.

## Materials and Methods

### Plasmid Construction

All genomic fragments in the *NBS1*-targeting constructs were amplified from DT40 genomic DNA using LA-PCR (Takara Bio, Kyoto) with the primers indicated below. To make the *NBS1Δ1–16* plasmid, the upstream and downstream arms were amplified with 5′-AGCGTCGACCCCGCGTATTTCAGCAGCCTG-3′ and 5′-AAAAGCTTTGGTTCCTCGGTGCTCCTCACC-3′ primers and 5′-ATCTGAAGCTTGCTCCACTGATATGTTTGC-3′ and 5′-AAGCGGCCGCTTTGTGATTCAAACACTGGA-3′ primers, respectively. The resulting amplified upstream fragment was cut at the *Not*I site (derived from genomic sequence) followed by Klenow treatment and subsequently a *Hin*dIII cut. The 2.5 kb blunt-end *Hin*dIII fragment was cloned into the *Xho*I (blunt ended with Klenow treatment) *Hin*dIII site of pBluescript II (Stratagene) (named the pBS/*NBS1* 5′ arm). Two oligonucleotides, containing either *Eco*RI-*Bam*HI-*Bgl*II-*Sal*I or *Bam*HI-*Bgl*II-*Hin*dIII, were inserted into the *Eco*RI-*Sal*I or *Bam*HI-*Hin*dIII site of the pBS/*NBS1* 5′ arm plasmid. The 3.5 kb 3′ arm was inserted into the *Hin*dIII-*NotI* site of the pBluescript (pBS/*NBS1* 3′ arm). To make the *NBS1Δ1–16* blasticidin (Bsr) gene-disruption construct, the Bsr^R^ marker cassette was cloned into the *Bam*HI site of the pBS/*NBS1* 5′ arm plasmid (with *Eco*RI-*Bam*HI-*Bgl*II-*Sal*I sites), followed by the ligation of the resulting plasmid (cut with *Sal*I and *Not*I) with the *Sal*I-*Not*I fragment containing the 3′ arm from the pBS/*NBS1* 3′ arm plasmid (*NBS1Δ1–16* Bsr). Similarly, a Puromycin- (Puro^R^) marker cassette was cloned into the *Bam*HI site of the pBS/*NBS1* 5′ arm plasmid, followed by the insertion of the *Hin*dIII and *Not*I fragment of the 3′ arm from the pBS/*NBS1* 3′ arm between the *Hin*dIII and *Not*I sites (*NBS1Δ1–16* Puro^R^). To make the *NBS1Δ13–16* gene-disruption construct, the upstream arm was amplified with 5′-TTGGAGGTCGACAAGCAAAACTGATGACGG-3′ and 5′-AAAGGATCCTCTTGGACAGCTGACAACCAG-3′ primers. The 7.5 kb *Sal*I- (in genomic sequence) *Bam*HI fragment of the amplified fragment was cloned into the *Xho*I-*Bam*HI site of the pBS/*NBS1* 5′ arm plasmid (named pBS/*Δ13–16* 5′ arm). A neomycin- (Neo) marker gene cassette was cloned into the *Bam*HI site of the pBS/*Δ13–16* 5′ arm. The resulting plasmid was ligated with the *Sal*I-*Not*I fragment of the 3′ arm used for the *NBS1Δ1–16* Bsr construct (*NBS1Δ13–16*). A probe for Southern hybridization was amplified from DT40 genomic DNA using the primers 5′-AAGCTTGCATGCAAACCTTGTTTTATCTTC-3′ and 5′-TGACTGCACTCTGCTCATTCTGGTATCTTC-3′.

The following two expression vectors were generated: 1) pBluescript-loxP-chicken β-actin promoter-multiple cloning site-internal ribosomal entry site (IRES) enhanced green fluorescent protein (EGFP) gene-loxP (named the plox vector), and 2) pBluescript-chicken β-actin promoter-multiple cloning site (named the pβ-actin vector). Chicken Nbs1^p95^ cDNA was amplified from pBS-*NBS1* by PCR with the 5′-AAGAATTCAGAAAGAACTAGAAGGTTAAG-3′ and 5′-TTTGGGCTCGAGTTACAGATCCTCTTCTGAGATGAGTTTTTGTTCTCTTCTCCTCTTCACATTAGG-3′ primers and cloned into the *Bgl*II-*Sal*I site of plox (plox/*NBS1^p95^*). To make the *NBS1^p70^* cDNA (Figure 2A), *NBS1^p95^* cDNA served as template DNA for PCR amplification using primers 5′-AAGGATCCATGGATGAGCCTGCCATTGG-3′ and 5′-TTTGGGCTCGAGTTAAGCGTAATCTGGAACATCGTATGGGTATCTTCTCCTCTTCACATTAGG-3′, and the amplified fragment was inserted into the *Bam*HI-*Not*I site of the pβ-actin plasmid (pβ-actin/*NBS1^p70^*).

Chicken Rad50 cDNA was amplified by a standard RT-PCR method with primers 5′-ATGGCCAAGATTGAGAAAATGAGCATCC-3′ and 5′-TTAATGAACGTATGAGCCAAGGGAGC-3′, and then cloned into pTRE2 (Clontech) (pTRE2/*RAD50*) (Accession #XM_414645). Two *RAD50* disruption constructs, *RAD50*-Bsr and *RAD50*-HisD, were expected to delete exon11 to 13 encoding amino-acid sequences from 579 to 735. The 3.9 kb 5′ arm was amplified from DT40 genomic DNA using primers 5′-TGCCATCAAGAGGAATCCAACTGGCCGTTA -3′ and 5′-CTCAGTGCTTTTGCCATGAAGCCAGTCTTC-3′ and cloned into pBluescript KS(+). The resulting plasmid was inserted with the 1.4 kb SpeI-SacI genomic fragment including exon 14, which was excised from a phage clone derived from the chicken genomic DNA library, where it served as the 3′ arm in the *RAD50* disruption construct. Lastly, marker cassettes, Bsr or HisD, were inserted into the *Bam*HI site to generate the *RAD50*-Bsr or *RAD50*-HisD gene-disruption construct. The genomic 3.4 kb *Sac*I-*Eco*RI fragment, which is located at downstream of the 3′ arm, was used as a probe for Southern-blot analysis.

### Cell Culture and DNA Transfection

Cells were cultured in RPMI1640, supplemented with 10^−5^ M β-mercaptoethanol, 10% fetal-calf serum and 1% chicken serum (Sigma, St Louis, MO) at 39.5°C. Methods for DNA transfection and genotoxic treatments are as described previously [77].

### Generation of *NBS1* Mutant Cells

*WT* DT40 cells were sequentially transfected with *NBS1Δ1–16* Bsr^R^ and subsequently with *NBS1Δ1–16-Puro^R^*-targeting constructs to obtain *NBS1^−/−/+^* cells. They were then transfected with an expression vector containing Cre-estrogen receptor chimeric recombinase (pANMerCreMer [46]) together with the plox/*NBS1* ^p95^ plasmid. The resulting *NBS1^−/−/+^/loxP-NBS1^p95^* cells were transfected with the *NBS1Δ13–16* gene-disruption construct to obtain *ΔNBS1/loxP-NBS1^p95^*. *ΔNBS1/loxP-NBS1^p95^* cells were transfected with the pβ-actin/*NBS1^p70^* vector to make *ΔNBS1/loxP-NBS1^p95^/NBS1^p70^* cells. *ΔNBS1/NBS1^p70^* cells were generated by exposing *ΔNBS1/loxP-NBS1^p95^/NBS1^p70^* cells to 100 nM tamoxifen for 3 days followed by subcloning, as described previously [46].

### Generation of *RAD50* Mutant Cells

*WT* DT40 cells were transfected with the *RAD50*-Bsr disruption construct to generate *RAD50^+/−^* cells. They were co-transfected with the *pTRE2/RAD50* and pTet-off (Clontech) plasmids simultaneously to make *RAD50* ^+/−^/*tetRAD50* cells. These cells were transfected with the *RAD50-HisD* construct to generate *RAD50* ^−/−^/*tetRAD50* cells. Conditional inactivation of the *RAD50* transgene was done using tetracycline as previously described [13].

### Measurement of Sensitivity of Cells to Killing by γ-Rays, Camptothecin, and Analyzes of Chromosome Aberrations

Clonogenic survival was monitored by a colony-formation assay, as described previously [77]. To measure sensitivity to camptothecin (Topogene, Columbus, OH), appropriate numbers of cells were plated into six-well cluster plates containing the complete medium and 1.5% methylcellulose (Aldrich, Milwaukee, WI), supplemented with camptothecin. Colony numbers were counted at 7 and 14 days, and the survival percentage was determined in terms of the number of colonies of untreated cells. To measure ionizing-radiation sensitivity, serially diluted cells were plated in the medium containing methylcellulose, irradiated with a ^137^Cs γ-ray source and then incubated. Measurement of chromosome aberrations was carried out as previously described [77].

### Western Blot Analysis

Methods described previously were used for the preparation of whole-cell extracts and western-blot analysis, with the following modifications. For western-blot analysis, the mouse monoclonal anti-human Nbs1 antibody (BD Transduction Laboratories catalog #611871) was used at a 1∶100 dilution, and HRP-conjugated donkey anti-mouse IgG antibody (Santa Cruz Biotechnology catalog #sc-2314) was used at a 1∶5000 dilution. Chicken Rad50 antiserum was raised in a rabbit against a whole protein of chicken Rad50. For the western-blot analysis, rabbit polyclonal anti-chicken Rad50 antibody was used at a 1∶100 dilution, and HRP-conjugated donkey anti-rabbit IgG antibody (Santa Cruz Biotechnology catalog #sc-2004) was used at a 1∶5000 dilution. For the western-blot analysis, rat monoclonal anti-mouse AID antibody (kindly provided by Dr. K. Kinoshita, Kyoto University) was used at a 1∶500 dilution, and HRP-conjugated donkey anti-rat IgG antibody (Jackson ImmunoResearch catalog #712-035-150) was used at a 1∶5000 dilution.

### Measurement of Targeted Integration Frequencies

To analyze the frequency of targeted integration events at the *OVALBUMIN* [78] and *HPRT* [79] loci, their disruption constructs were transfected into cells. Following selection of clones resistant to appropriate antibiotics, Southern-blot analysis was performed.

### Analysis of Rate of sIgM Gain and Loss

We confirmed that *ΔNBS1/NBS1^p70^* cells retained the same frame-shift mutation in the V sequence as do *WT* cells [21]. Generation frequency of surface IgM (sIgM) loss variants as well as sIgM-gain revertants were monitored by flow-cytometric analysis of cells that had been expanded for 3 weeks after subcloning and then stained with fluorescein isothiocyanate-conjugated (FITC) goat anti-chicken IgM (Bethyl, Montgomery, TX). At least 30 subclones were analyzed in each genotype. To enhance Ig gene conversion, trichostatin A (TSA, Wako Osaka, concentration: 1.25 ng/ml) was added to a mixture of sIgM-negative subclones from *WT* and the *ΔNBS1*/*NBS1^p70^* #1 genotypes shown in Figure 3B. The fraction of sIgM^+^ revertants was monitored over time, as described previously [55]. In each analysis, the abundance of sIgM-positive cells was determined as the percentage of live cells whose FITC fluorescence fell at least eight fold more than the FITC fluorescence peak of sIgM negative cells. Ig gene conversion frequency of unsorted cells was calculated based on the number of gene-conversion events, of analyzed V_λ_ clones and of cell divisions.

### Generation of AID, Chicken Exo1, and SbcB Expression Retrovirus and Infection into DT40 Cells

For retrovirus infection, the pMSCV-IRES-GFP recombinant plasmid was constructed by ligating the 5.2 kb *Bam*HI-*Not*I fragment from pMSCVhyg (Clontech) with the 1.2 kb *Bam*HI-*Not*I fragment of pIRES2-EGFP (Clontech). Mouse AID [58] or chicken ExoI (Accession #AB084249) or SbcB cDNA was inserted between the *Bgl*II and *Eco*RI sites of pMSCV-IRES-GFP [58]. The preparation and infection of retroviruses were carried out as previously described [58]. Expression of the GFP was confirmed by flow cytometry. The efficiency of infection was more than 90%m as judged by GFP expression. Cells were sub-cloned into 96 well-plates a day after infection. After 2 weeks, clones displaying a bright GFP signal were determined by FACS analysis.

### Generation of Human TdT-Expressing Clones and Analysis of Ig Sequence Diversification

*WT* DT40 cells were transfected with a pSV2neo-based plasmid containing human TdT under control of the β-globin promoter and IgH enhancer [67] by electroporation as previously described. Clones were analyzed for TdT expression by indirect immunofluorescence microscopy using a mouse monoclonal anti-TdT (Dako) followed by anti-mouse Igκ conjugated to FITC. TdT-positive clones were expanded for 4 weeks, following which Ig-negative loss variants were sorted by FACS and the rearranged light-chain gene sequenced and analyzed as previously described [20].

### Analysis of Ig V_λ_ Nucleotide Sequence

DNA was extracted from three to five clones from genotypes at 14 days after AID, Exo1 or SbcB retrovirus infection, or at 28 days after TSA treatment. PCR-amplified fragments of V_λ_ segments were cloned into a plasmid and subjected to base-sequence analysis. Rearranged V_λ_ was amplified by PCR with Pyrobest DNA polymerase (Takara Bio) (30 cycles of 94°C for 30 s, 60°C for 1 min, and 72°C for 1 min) with 5′-CAGGAGCTCGCGGGGCCGTCACT-GATTGCCG-3′ and 5′-GCGCAAGCTTCCCCAGCCTGCCGCCAAGTCCAAG-3′ primers, as previously described [20]. PCR products were cloned into the TOPO pCR2.1 cloning vector (Invitrogen) and sequenced with the M13 forward (−20) or reverse primer using an ABI PRISM 3100 sequencer (Applied Biosystems). Sequence alignment using GENETYX-MAC (Software Development, Tokyo, Japan) allowed the identification of changes from the parental sequences in each clone. Differentiating between non-templated nucleotide substitutions and gene conversion was carried out as previously described [20]. The rate of hypermutation was calculated based on mutation frequency and number of cell divisions (42 cycles in *WT* and 37 cycles in *ΔNBS1*/*NBS1^p70^* for 14 days).

### I-*Sce*I-Induced Gene Targeting

10^7^ cells were suspended in 0.1 ml Nucleofector Solution T (amaxa), and electroporated using a Nucleofector (amaxa) at program B-23. 2 µg of linear *3′ neo* DNA and 2 µg of circular I-*Sce*I expression vector (pcBASce), together with 2 µg of either control (pBluescript II KS+), SbcB or chicken Exo1 expression vector, were transfected. *3′ neo* DNA was amplified by PCR from the *SCneo* neo substrate plasmid [69] using Phusion DNA polymerase (Finnzymes) (30 cycles at 94°C for 30 s, 60°C for 30 s, and 72°C for 2 min), with 5′-GGATCGGCCATTGAACAAGATGGATTGCAC-3′ and 5′-GGAAACAGCTATGACCATGATTACGCCAAG-3′ primers. The amplified fragment was used for electroporation, as previously described [44]. 24 hours after electroporation, the number of live cells was counted by FACS and transferred to 96 well-cluster trays with or without 2.0 mg of G418 per ml. Cells were grown for 7 days, and HR frequencies were calculated by the following equation: HR frequency (colonies/cell) = number of G418-resistant colonies/(plating efficiency of transfected cells in the absence of G418×number of live cells determined by FACS at 24 hour after electroporation) [44].

## Supporting Information

Figure S1 *RAD50* gene-disrupted mutants are lethal to cells. *RAD50^−/−^* cells were conditionally created using the tet-repressible promoter (*RAD50^−/−^*/*tetRAD50*), as described for the generation of *MRE11*-deficient DT40 cells [13]. (A) Schematic representation of a part of the *RAD50* locus, the gene disruption constructs and the configuration of the targeted alleles. Solid boxes indicate the position of exons. Only disrupted exons are indicated. Relevant *Eco*RI sites and the position of the probe used in Southern-blot analysis are indicated. (B) *RAD50* gene-targeting was confirmed by Southern-blot analysis. *Eco*RI-digested genomic DNA from cells with the indicated genotypes of the *Rad50* gene was analyzed, using the probe shown in (A). Positions of hybridizing fragments of the *WT* and targeted loci are indicated. (C) The *tetRAD50* transgene expression was inhibited by the addition of doxycycline (Dox). Rad50 protein levels were reduced by at least 100 fold 24 hours after the addition of doxycycline. (D) Growth curves corresponding to the indicated cell cultures. Experiments were done at least three times. +Dox represents continuous exposure to doxycycline. The cells ceased to proliferate four days after the addition of Dox and eventually all died, indicating that *RAD50* plays an essential role in the cellular proliferation of any vertebrate cell. (E) Chromosomal breaks accumulated in Rad50 depleted cells. *RAD50* ^−/−^ cells were exposed to Dox for six days. Fifty mitotic cells were analyzed for each chromosomal analysis.
(3.1 MB EPS)
